# Supplementary material for: Developing functional markers for vitamin E biosynthesis in oil palm
Source: PLoS One. 2021 Nov 19;16(11):e0259684. doi: 10.1371/journal.pone.0259684 (PMC8604351; doi:10.1371/journal.pone.0259684)
Supplement: S1 Raw images — (PDF) [file pone.0259684.s008.pdf]

S1_raw_images

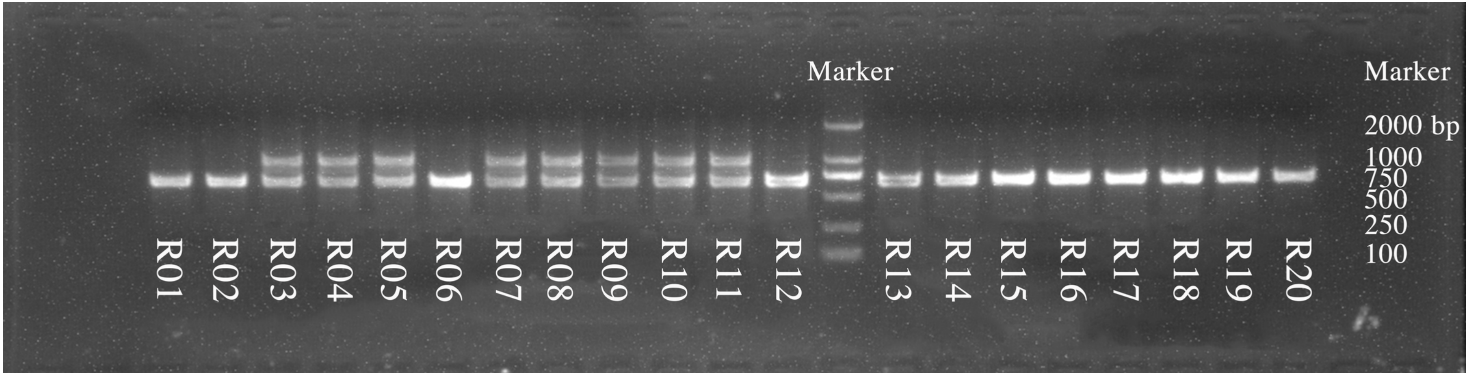

Annotation: This is the original image of Fig 5A. The image was captured by using G:BOX F3 Gel Documentation System (Syngene, UK).
